# A Data-Driven Social Network Intervention for Improving Organ Donation Awareness Among Minorities: Analysis and Optimization of a Cross-Sectional Study

**DOI:** 10.2196/14605

**Published:** 2020-01-14

**Authors:** Michael Douglas Murphy, Diego Pinheiro, Rahul Iyengar, Gene Lim, Ronaldo Menezes, Martin Cadeiras

**Affiliations:** 1 Department of Medicine University of California, Los Angeles Los Angeles, CA United States; 2 Department of Internal Medicine University of California, Davis Sacramento, CA United States; 3 Mav12 Inc Santa Monica, CA United States; 4 Department of Computer Science University of Exeter Exeter United Kingdom

**Keywords:** organ donation, social media, minority health, community health education

## Abstract

**Background:**

Increasing the number of organ donors may enhance organ transplantation, and past health interventions have shown the potential to generate both large-scale and sustainable changes, particularly among minorities.

**Objective:**

This study aimed to propose a conceptual data-driven framework that tracks digital markers of public organ donation awareness using Twitter and delivers an optimized social network intervention (SNI) to targeted audiences using Facebook.

**Methods:**

We monitored digital markers of organ donation awareness across the United States over a 1-year period using Twitter and examined their association with organ donation registration. We delivered this SNI on Facebook with and without optimized awareness content (ie, educational content with a weblink to an online donor registration website) to low-income Hispanics in Los Angeles over a 1-month period and measured the daily number of impressions (ie, exposure to information) and clicks (ie, engagement) among the target audience.

**Results:**

Digital markers of organ donation awareness on Twitter are associated with donation registration (beta=.0032; *P*<.001) such that 10 additional organ-related tweets are associated with a 3.20% (33,933/1,060,403) increase in the number of organ donor registrations at the city level. In addition, our SNI on Facebook effectively reached 1 million users, and the use of optimization significantly increased the rate of clicks per impression (beta=.0213; *P*<.004).

**Conclusions:**

Our framework can provide a real-time characterization of organ donation awareness while effectively delivering tailored interventions to minority communities. It can complement past approaches to create large-scale, sustainable interventions that are capable of raising awareness and effectively mitigate disparities in organ donation.

## Introduction

### Background

Organ transplantation is the therapy of choice for patients with end-stage organ failure. Over the past three decades, organ transplantation has saved more than 2 million life-years in the United States alone [1]. However, only half of US adults are registered as organ donors [2], and the current pool of recovered organs inadequately meets the particular medical demand of patients from ethnoracial minorities [3]. With the current shortage of organ donors and an ever-increasing incidence of end-stage organ failure, the number of patients left in need of organ transplantation has grown: only 1 out of 4 patients on the organ wait list will eventually receive the organ transplant needed [4,5]. The success of organ transplantation depends on the patient’s histocompatibility with the donated organ, which reaches higher similarities with donors from comparable ethnoracial communities [6,7]. However, the current pool of available organs mainly consists of organs from nonminority donors because of the disproportionate scarcity of ethnoracial minority donors [8]. Increasing the general number of organ donors can mitigate the overall organ shortage, but we can only effectively address the disproportional need of patients from underrepresented demographics by specifically increasing the number of ethnoracial minority donors.

The lack of minority organ donors is generally attributed to insufficient health literacy, which affects how individuals make educated health decisions about their lives and the lives of their families and overall community [9-12]. In the case of organ donation, health literacy specifically impacts the likelihood of individuals to register as organ donors and to consent for the organ donation of their relatives [13,14]. Given that individuals from minority communities tend to have lower health literacy than their ethnoracial majority counterparts, these communities have a relatively lower likelihood of registering as organ donors [9,15,16]. To effectively address this disparity, we need to raise awareness among individuals from minority communities by supplementing them with tailored educational materials about organ donation.

Educational interventions such as the National Minority Organ Tissue Transplant Education Program have generated large-scale and sustainable change across minority communities by raising health literacy [3,14,17-19]. Sustainable large-scale diffusion of health education mainly depends on individual willingness to disseminate the health education received within their social network and how well an individual’s social network is integrated within the relevant social constructs as a whole [20,21]. Individuals are more willing to disseminate educational content that is socioculturally tailored and content that is being already disseminated via existing social ties, including family, friends, and other individuals within their community [22,23]. To reach and increase the willingness of individuals from minority communities, health care professionals have created community-based interventions by targeting individuals within these communities with educational content that is socioculturally tailored [3,13,14]. Naturally, a community-based intervention indirectly targets individuals who are likely to be socially connected and, thus, can independently reinforce the dissemination of educational content through social ties. Therefore, community-based interventions not only reach individuals from minority communities but potentiate sustainable change. However, minority communities are not just unintegrated from the whole social system but also isolated from each other, making these traditional interventions ineffective in diffusing health education among these communities at a large scale [3,14].

### Objectives

Web-based social networking platforms, also known as social media (eg, Twitter and Facebook), have been proposed as modern venues for the cost-effective delivery of large-scale health interventions with higher outreach in domains as diverse as physical activities, smoking cessation, weight loss, and mental health [20-25]. As social media can be a proxy for real social networks [26], social media platforms are exceptionally suitable for health interventions in which the implicated spreading phenomena are mainly driven by social mechanisms [10,27-29] and can facilitate the delivery of *network interventions* [23]. Network interventions foster higher cascades of behavioral health changes by leveraging the network structure underlying the social context of targeted individuals [30,31]. For instance, the simple decision to register for an internet-based health forum can involve a complex contagion in which individuals require independent social reinforcement and are more susceptible to change their behavior as more peers change theirs [22].

Previous studies have demonstrated the potential of social media to enhance organ donation by promoting health awareness and increasing the number of donor registration rates among minorities [13,32]. However, we still lack a comprehensive framework that allows us to effectively monitor and deliver large-scale network-based interventions of health literacy in real time. We proposed a data-driven framework for improving organ donation awareness by monitoring awareness regarding organ donation and delivering an optimized social network intervention (SNI) using 2 distinct social media interfaces: Twitter for monitoring and Facebook for intervention. Using our framework, we monitored awareness about organ donation over 1 year, then developed and implemented an SNI for improving awareness among minorities over 1 month. The results suggested that our framework can provide a real-time characterization of awareness about organ donation while optimizing the delivery of SNIs to individuals from minority communities. Our data-driven framework has the potential to effectively create large-scale and sustainable interventions to improve organ donation awareness among minorities.

## Methods

### Identification of Structural Disparities in Organ Donation

To structurally assess disparity, we modeled the connectivity between organ donors and transplant recipients with geographical social networks (GSNs) using the United Network for Organ Sharing (UNOS) database [29]. This dataset includes approximately 438,000 organ transplants conducted in the United States between 1987 and 2010 containing clinical, geographic, and social information about donors and recipients. In our GSN, nodes are home locations of donors or recipients at the zip code level, and links are organ transplants that were recovered from organ donors living at the origin node and transplanted into recipients living at the destination node. We built a separate ethnoracial GSN for Hispanics, blacks, and whites, focused on recipients [29]. For instance, in the white GSN, the destination node of every link is the home address of a white recipient, whereas the origin node can be the home address of donors from any race/ethnicity. Note that origin nodes (ie, home address of donors) can also be destination nodes (ie, home address of recipients). Finally, we had 3 ethnoracial GSNs that represent the structure of the organ transplantation flow for each race/ethnicity.

Using network science [31-34], we compared our GSNs by quantifying the local and global connectivity according to GSN-respective clustering coefficients and the average path lengths. The clustering coefficient () quantifies the likelihood of 2 nodes being connected, given they share a common node, ranging from 0 (ie, low clustering) to 1 (ie, high clustering). For instance, in a social network of friendships, a clustering coefficient can quantify how likely my friends are also friends with one another. In our GSN, this measure quantifies how likely organ transplants occur between home addresses A and B given that they occur from home address C to both home addresses A and B. This measure of clustering between nodes within a single local network is an influential factor in ascertaining network shortcomings or structural disparities, which could lead to unequal access to donor organs.

Similarly, the average path length (*L*) is a global measure of connectivity, and it quantifies the typical number of links connecting 2 nodes in the whole network, ranging from 1 to the diameter of the network (ie, the shortest to longest path length between 2 nodes). In a social network of friendships, for instance, the average path length quantifies how many friends typically separate 2 individuals. In our GSN, the average path length quantifies the number of links that typically separate any 2 home addresses among which organ transplants are occurring. This measure of relative accessibility among connected nodes within a global network is an influential factor in uncovering structural disparities, which could lead to strained or unsuitable access to donor organs [31-33].

Finally, we also identified the communities of home addresses with similar organ transplantation dynamics within each ethnoracial GSN using community detection [34,35]. For each network, we measured the number of nodes (*N_n_*), links (*M*), average degree (*M/N_n_*), clustering coefficient (*CC*), average path length (*L*), and the number of communities (*N_c_*). Owing to the underlying network of organ transplantation flow, the connectivity measures along with the number of communities attempt to assess the structural disparity in organ transplantation. 

### Digital Sensor for Organ Donation Awareness in Social Media

In past work, we have explored the extent to which social media (ie, Twitter) can be used as a sensor for organ donation awareness [28,36]. Twitter is a convenient tool for real-time social sensing because it allows for data collection from most of its users as long as these users set their profile as public. We demonstrated that Twitter has sufficient information regarding organ donation awareness and has the potential to be employed as a social sensor for organ donation campaigns by characterizing conversations according to the volume of mention to different solid organs [28,36].

The organ-related tweets were automatically collected using the minimalist Twitter application programming interface (API) for Python [37], which searches the Twitter stream API, constraining the search by filtering the tweets containing a predefined set of organ donation digital markers among the 140 characters of the tweet text. Organ donation digital markers were defined based on a set of 5 context words (ie, transplant, transplantation, donor, donation, and donate) and a set of 6 subject words (ie, heart, kidney, liver, lung, pancreas, and intestine). For the subject words, only the 6 major solid organs were included, and other possible subject words such as cornea, bone, and skin were not considered. This approach ensures that each collected tweet contains at least one of the 5 words from the context set and at least one of the 6 words from the subject set. Besides, it also ensures that the individuals who wrote these tweets are aware of at least one aspect of organ donation.

Each collected tweet was subsequently augmented with its user’s location. Only 0.49% (4875/975,021) of tweets contained the global positioning system coordinates from where the tweet was posted. Therefore, a structural address containing the country, state, county, city, and zip code was automatically extracted from the self-reported location contained in the user profile using the python package geopy and the Nominatim search engine for OpenStreetMap data [38]. Finally, augmented tweets were filtered to only retain those belonging to US users. Therefore, our final tweet dataset was conceived in the context of organ donation and included 1 year of data representing more than 70,000 users in the United States.

### Calibration and Efficacy of the Digital Sensor

To validate the extent to which the organ-related tweets collected using Twitter could be used as a digital sensor for organ donation awareness in social media, we assessed the association between the number of organ-related tweets collected by the digital sensor and the number of organ donor registrations. The data of organ donor registrations were obtained from Donate Life California [39]. It contains donor registrations at the zip code level from Los Angeles county. Owing to the scarcity of tweet data at the zip code level, the number of organ-related tweets and donor registrations were both subsequently aggregated at the city level. Afterward, a Poisson regression model was used to model the number of organ donor registrations as a function of the number of organ-related tweets and the size of the population at the city level. A data-intensive approach was used as a second independent model for validating the consistency of the Poisson regression model. The data-intensive approach grouped cities into 4 groups of incremental tweet rate percentile intervals: 0-25, 25-50, 50-75, and 75-100. Afterward, for each group, it estimates the organ donor registration rate using 10,000 bootstrap samples with replacement.

### Digital Intervention Using the Facebook Advertising Platform

Our intervention consisted of targeting Facebook users with educational materials about organ donation via Facebook’s advertising platform. Our content comprised short motivational videos associated with testimonials, current facts, and statistics about organ donation, as well as a link to the organ donation registration website (Donate Life California, Sacramento, California) [40]. All text and content used as educational content for the intervention was developed in collaboration with One Legacy, an organ procurement organization (OPO) for Southern California. OPOs follow the best practices in the development of material for organ donation, which is guided by diverse and multidisciplinary focused groups.

Using Facebook’s advertising platform from August 4 to September 3, 2016, we systematically targeted communities found to be at risk for a structural disparity. The criteria were based on location, sex, age, and income level, and thus, the intervention was delivered to a selected audience instead of a mass of incidental recipients. Targeting implicated individuals, such as in community-based interventions, can improve the intervention effectiveness because it increases the likelihood of targeting connected individuals who in turn are more likely to act as social reinforcers for others [41]. Targeting these connected individuals also facilitates the creation of organic sustainability by the mechanisms of engagement existing on Facebook (eg, like and share) [41,42]. After our intervention initially exposes educational content to targeted users on Facebook, these users can actively disseminate the targeted content among their social network and, thus, contribute to the exposure of these contents to other individuals who were not previously targeted by the intervention in the first place [20]. This additional organic exposure is ultimately controlled by Facebook’s algorithm, which is inherently biased toward targeting these exposures to similar users.

### Measure Effect and Optimization

The number of impressions (*I*), clicks (*C*), and page views (*V*) were used to measure the effectiveness of our SNI. These measurements were provided daily by the Facebook advertising platform throughout the intervention. Our SNI delivered content in 2 phases: pre optimization and post optimization. In the preoptimization phase, from August 4 to August 23, the SNI delivered all content with equal proportion and calculated the number of clicks per impression (*C/I*) associated with each content. At the end of the preoptimization period, the SNI learned which content had the highest capability of fostering active engagement among the target audience as measured by the content’s *C/I* ratio. Afterward, in the postoptimization phase, from August 24 to September 3, the intervention was optimized to deliver the educational content associated with the highest *C/I* ratio. Given the absence of a baseline, we used the optimization as an instrumental variable and considered the intervention before optimization as a control group for the intervention after optimization. Ordinary least squares (OLS) regression was used to model the number of clicks per impression (*C/I*) as a function of both the number of impressions (*I*) and the use of optimization (*O*). The optimization was outsourced to the company MAV 12. 

**Figure 1:**
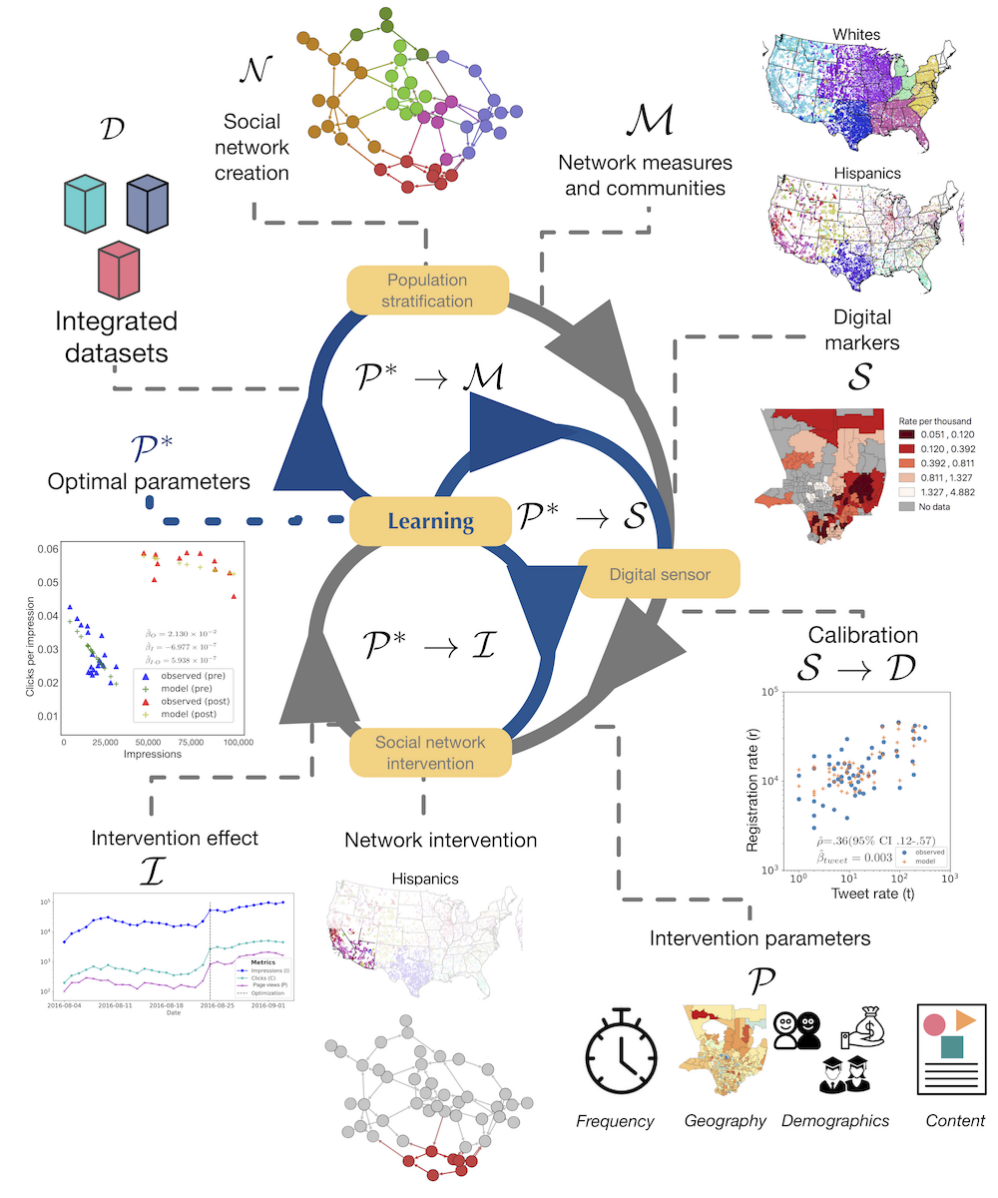
Conceptual framework of the optimized social network intervention.

The overall framework of the SNI is summarized in Figure 1. To characterize structural disparity, separate ethnoracial network-based community analysis was performed. To characterize population awareness about organ donation, data mining of the digital markers of organ donation awareness was performed using Twitter and subsequently calibrated using organ donation registrations. Educational content was delivered to the targeted audience using Facebook in 2 phases: preoptimization and postoptimization. In the preoptimization phase, the SNI delivered all contents and calculated the number of clicks per impression associated with each content. At the end of the preoptimization period, the SNI learned which content had the highest rate of clicks per impression. Afterward, in the postoptimization phase, the intervention was optimized to deliver the educational content associated with the highest rate of clicks per impression.

In general, the SNI characterizes the communities within the transplantation system using network analysis and monitoring the digital markers of organ donation awareness using Twitter. The calibration of these markers on Twitter was performed in conjunction with existing datasets of donation registration from Donate Life, which substantiated the delivery of a large-scale SNI using Facebook. Real-time data were collected to uncover the optimal content for user engagement, which allowed us to optimize the intervention to better target the intended demographics. The University of California Los Angeles Investigational Review Board approved the study.

## Results

### Assessment of Disparities in Organ Donation

Each of the ethnoracial GSNs focused on organ transplant recipients elucidates both local and global measures of connectivity as well as the varying number of ethnoracial communities within the whole social system (Table 1). The Hispanic GSN has an average degree (*M/N_n_*) that indicates that Hispanic recipients typically receive organs from a fewer number of distinct donor addresses. Furthermore, the Hispanic GSN had the highest average path length (*L*), which indicates that Hispanic recipients receive organs from donors living further away in their social network. In addition, the Hispanic GSN is divided into a greater number of communities (*N_c_*) when compared with the white GSN, which can indicate a distinct structural disparity in the ethnoracial pattern in the flow of organs, which needs to be addressed.

**Table 1:** Network measures of ethnoracial geographic social network focused on recipients.

GSN^a^	*N_n_* ^b^	*M* ^c^	*M/N_n_* ^d^	*CC* ^e^	*L* ^f^	*N_c_* ^g^
All	31,793	266,812	17	0.068	3.968	9
Hispanic	12,025	31,232	5	0.092	5.166	11
Black	16,925	53,697	6	0.126	4.738	12
White	29,606	172,506	12	0.044	4.284	6

^a^GSN: geographical social network.

^b^*N_n_*: number of nodes.

^c^*M*: links.

^d^*M/N_n_*: average degree.

^e^*CC*: clustering coefficient.

^f^*L*: average path length.

^g^*N_c_*: number of communities.

**Figure 2:**
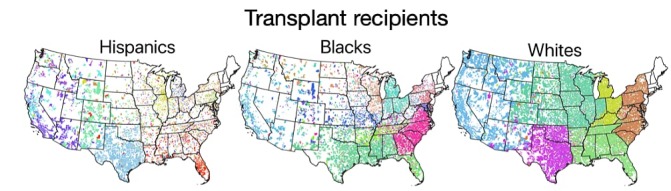
Ethnic/racial communities of geographic social network (GSN). The communities are extracted from separately generated GSNs from transplant recipients that are Hispanics (left), blacks (center), and whites (right). Minority populations (ie, Hispanics and blacks) experience a greater number of disorganized communities within the United States.

By examining the geographic spread of these communities across the United States (Figure 2), one can see that Hispanic communities appear more geographically spread. Although similar white communities are located close to one another and thus form well-defined geographic boundaries, Hispanic communities are more geographically dispersed such that same communities have a higher chance of being located far from each other. This higher geographic spread of communities in the Hispanic GSN along with its higher average path length quantitatively describes unintended differences in the organ allocation mechanism for Hispanic recipients. In principle, organ allocation should be as local as possible according to UNOS.

### Evaluation of a Sensor of Organ Donation Using Twitter

**Figure 3:**
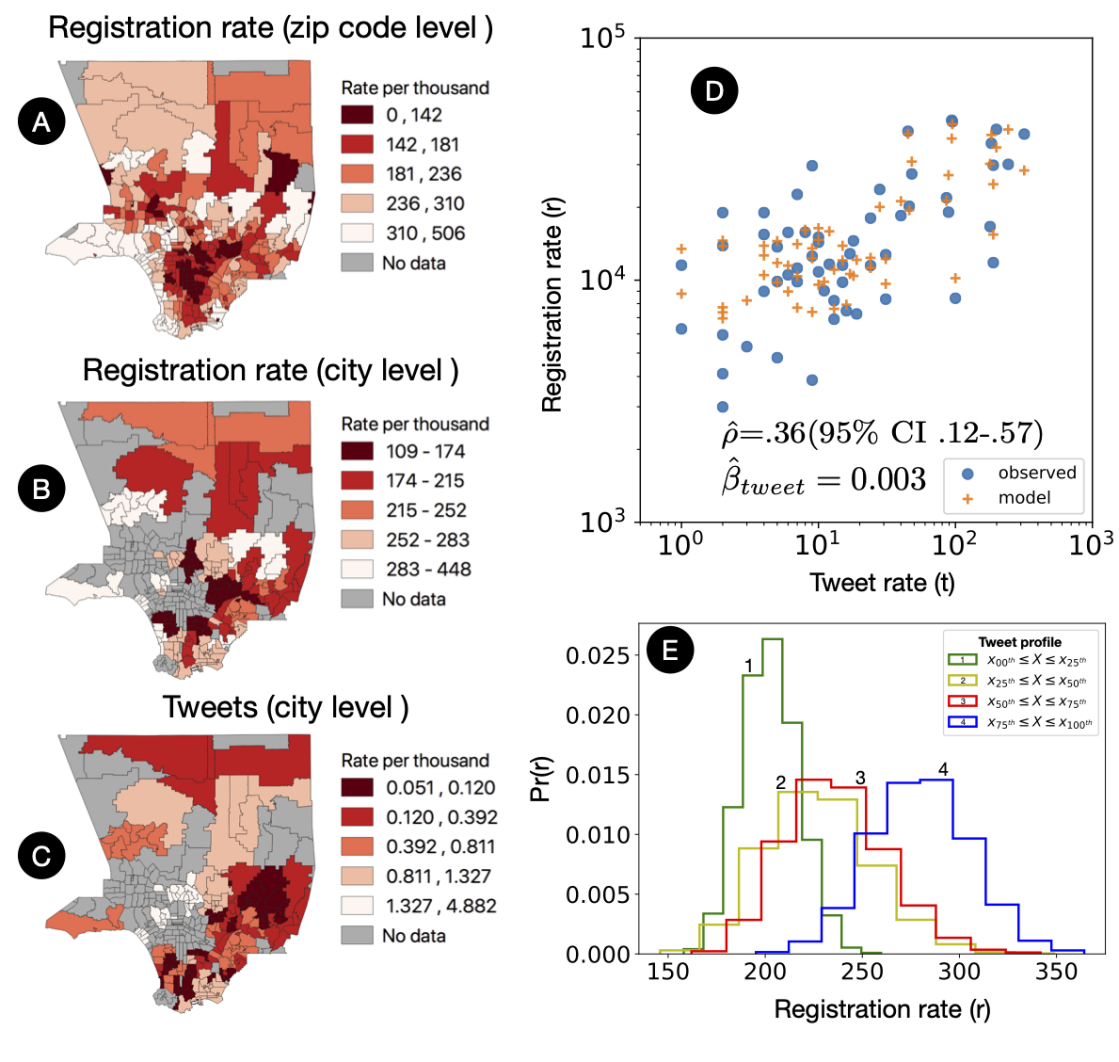
Association between organ-related tweets and organ donation registrations. (A) Organ donation registrations at the zip code level. (B) Organ donation registrations aggregated at the city level. (C) Organ-related tweets at the city level. (D) Poisson model of donation registration predicted by organ-related tweets after controlling for population size. (E) The profile of organ-related tweet percentile of a city is associated with the organ donation registrations of that city.

The descriptive statistics of our collected tweets are described in Table 2. Tweets were collected using our real-time organ donation sensor, and organ donation registrations were obtained from the Department of Motor Vehicles in the greater Los Angeles Area (LA County). Our organ donation sensor shows that the number of organ-related tweets are associated with the number of organ donation registrations (Figure 3). After normalizing for the population size, the number of organ donor registrations (Figure 3) are significantly correlated with the number of organ-related tweets at the city level (Figure 3). A Poisson regression predicts that each 10 additional organ-related tweets are associated with a 3.20% (33,933/1,060,403) increase in the number of donor registrations (Figure 3). Similarly, the data-intensive bootstrapping predicts that, on average, the number of organ donor registrations can vary (Figure 3) from 202 (95% CI 176-32) for cities with organ-related tweet rates between 0 and 25 percentiles to 279 (95% CI 231-329) for cities with organ-related tweet rates between 75 and 100 percentiles.

Yearly state-level organ registration data obtained from publicly available Donate Life annual reports from 2009 to 2016 [2] were additionally used to validate that the organ-related tweets collected in 2016 and further aggregated at the state level increasingly correlate with more recent registration data. For instance, organ-related tweets are more correlated with 2016 registrations (*r*=.81; *P*<.01) than with 2009 registrations (*r*=.51; *P*<.01) and 2012 registrations (*r*=.70; *P*<.01).

**Table 2:** Descriptive statistics of tweets collected by the organ donation Twitter sensor.

Statistic	Value
Data collection start date	April 22, 2015
Data collection end date	May 11, 2016
Data collection number of days	385
Number of collected tweets	134,986
Number of Twitter users	71,947
Average number of tweets per day	350
Average number of tweets per user	1.88
Number of organs mentioned per tweet	1.03
Number of organs mentioned per user	1.13

### Exposure to a Focused Audience

The SNI reached more than 1 million individual users on Facebook (Table 3). Users in social media, including Facebook, can be overrepresented or underrepresented when compared with the actual population. As the targeted audience is increasingly narrowed, such deviation can be intensified. The advertising platform on Facebook provides insights on the targeted audience according to multiple criteria, including gender and socioeconomics (Table 3). For instance, the audience targeted by our SNI had moderately lower household income. However, more women (939,666/1,174,583; 80.00%) were unexpectedly reached than men (234,917/1,174,583; 20.00%).

The educational content associated with the highest clicks per impression (*C/I*) during the first phase of the intervention is defined as the most appealing content. Such content is subsequently used to optimize the intervention in a second phase. This optimization played a key role in exposing the most appealing content to the targeted audience while promoting higher engagement rates per impression.

**Table 3:** Population demographics targeted by the social network intervention. Overall, the audience targeted by our SNI had moderately lower household income, and more women were reached than men.

Demographic characteristics		Values (n=1,174,583)
**Gender, n (%)**		
	Women	939,666 (80.00)
	Men	234,917 (20.00)
**Age (women), n (%)**		
	18-24	58,729 (5.00)
	25-34	293,646 (25.00)
	35-44	293,646 (25.00)
	45-54	234,917 (20.00)
	55-64	176,187 (15.00)
	>65	58,729 (5.00)
**Age (men), n (%)**		
	18-24	0 (0.00)
	25-34	411,104 (35.00)
	35-44	411,104 (35.00)
	45-54	411,104 (35.00)
	55-64	0 (0.00)
	>65	0 (0.00)
**Household income (USD), n (%)**		
	30-40	117,458 (10.00)
	40-50	176,187 (15.00)
	50-75	411,104 (35.00)
	75-100	176,187 (15.00)
	100-125	117,458 (10.00)
	125-150	117,458 (10.00)
	150-250	117,458 (10.00)
	250-350	0 (0.00)
	350-500	0 (0.00)
	>500	0 (0.00)
**Household ownership, n (%)**		
	Renter	352,375 (30.00)
	Owner	822,208 (70.00)

### Efficacy of Exposure and Engagement

**Figure 4:**
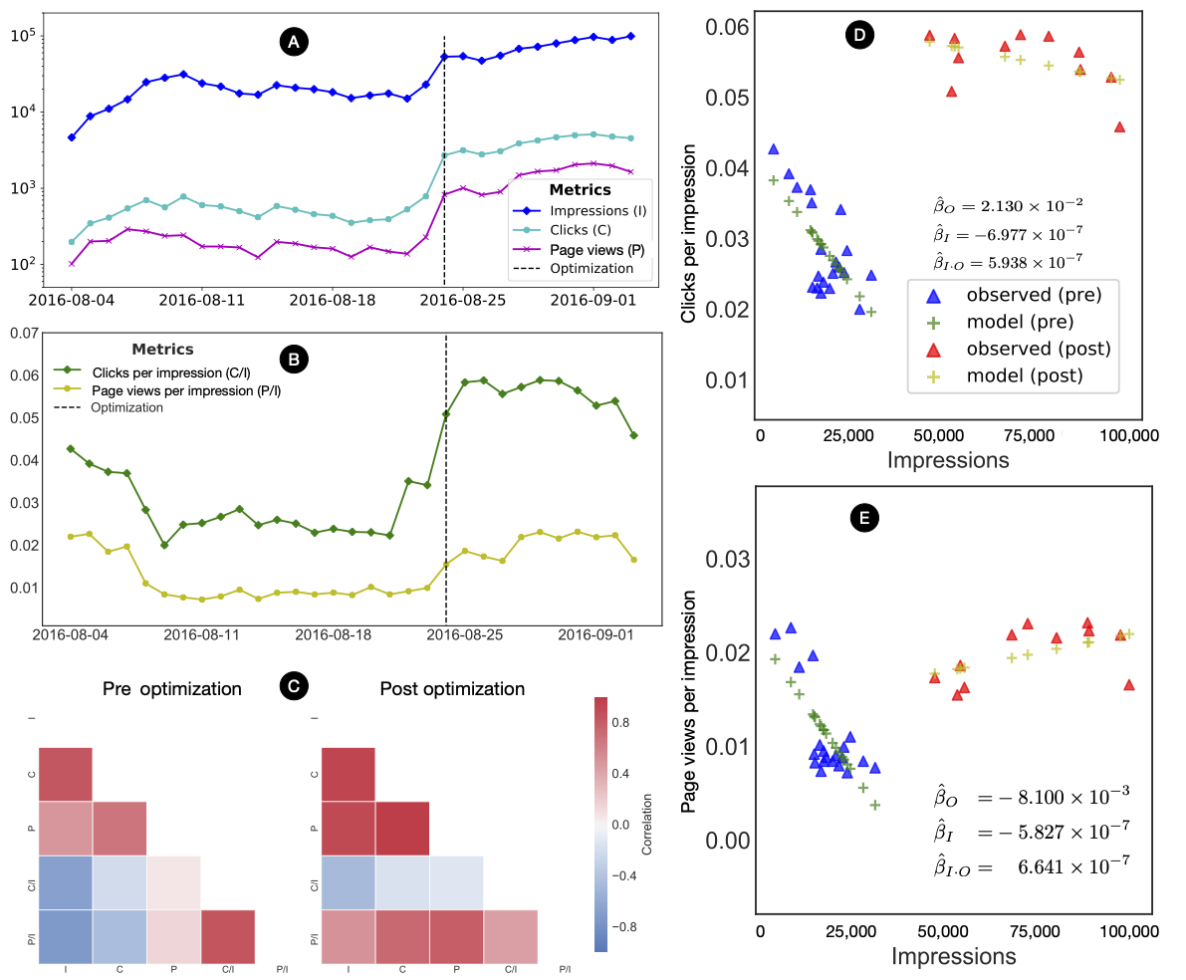
Effectiveness of the social network intervention. (A-B) The daily metrics of the impressions, clicks, page views, as well as their normalized versions, clicks per impression, and page views per impression. (C) The rate of clicks per impression and page views per impression became more positively associated after the optimization. (D-E) The regression analysis implicates the use of optimization plays a key role in positively affecting clicks per impression and page views per impression. For instance, after the optimization, clicks per impression was 0.0213 higher.

Facebook’s advertisement platform provided the number of impressions, clicks, and page views daily (Table 4; Figure 4). These measurements are highly correlated, and this high correlation structure increased after optimization (Figure 4). To control for differences between resource utilization after the optimization as measured by the number of impressions, the number of clicks (*C/I*) and page views (*V/I*) were normalized by the number of impressions (Figure 4). Although *C/I* and *V/I* are negatively correlated with before the optimization, both ratios become more positively correlated after the optimization (Figure 4).

The results of the OLS regression indicate that the use of optimization can increase *C/I* (beta=.0213; *P*<.004). For instance, 21,000 clicks can be additionally fostered when exposing 1 million individuals (Table 5 and Figure 4). According to the regression, an additional 21 (95% C 8-35) clicks can be obtained per thousand of impressions after the optimization, with the number of clicks per thousand impressions increasing from 42 (95% CI 35-48) to 63 (95% CI 50-77). One can see a saturation between clicks and impressions. The *C/I* began to saturate as *I* increased, but this saturation was lower after the optimization. Before the optimization, as *I* increased, *C/I* decreased from 41 (95% CI 40-41) to 21 (95% CI 10-31). This saturation vanished after the optimization, and *C/I* has not statistically changed as *I* increased. Conversely, *V/I* was not significantly changed after the optimization. All data can be made available for future studies upon request.

**Table 4:** Social network intervention before and after optimization. The number of impressions, clicks, and page views provided daily by Facebook’s advertisement platform.

Date/period		*I* ^a^	*C* ^b^	*V* ^c^	*C*/*I*^d^ (%)	*V*/*I*^e^ (%)
**All intervention**						
	Total period	1,174,583	53,988	19,901	4.60	1.69
**Pre optimization**						
	Total period	372,524	10,077	3705	2.71	0.99
	August 4	4639	198	102	4.27	2.20
	August 5	8831	346	200	3.92	2.26
	August 6	11,058	412	204	3.73	1.84
	August 7	14,731	544	290	3.69	1.97
	August 8	24,697	699	272	2.83	1.10
	August 9	28,165	563	237	2.00	0.84
	August 10	31,336	778	242	2.48	0.77
	August 11	23,904	602	172	2.52	0.72
	August 12	21,661	578	172	2.67	0.79
	August 13	17,584	501	167	2.85	0.95
	August 14	16,884	417	124	2.47	0.73
	August 15	22,518	585	198	2.60	0.88
	August 16	20,854	523	188	2.51	0.90
	August 17	19,964	458	168	2.29	0.84
	August 18	18,252	435	161	2.38	0.88
	August 19	15,264	353	126	2.31	0.83
	August 20	16,552	381	168	2.30	1.02
	August 21	17,594	392	148	2.23	0.84
	August 22	15,061	528	138	3.51	0.92
	August 23	22,975	784	228	3.41	0.99
**Post optimization**						
	Subtotal	802,059	43,911	16,196	5.47	2.02
	August 24	53,280	2708	825	5.08	1.55
	August 25	54,076	3154	1007	5.83	1.86
	August 26	47,259	2778	819	5.88	1.73
	August 27	55,165	3067	898	5.56	1.63
	August 28	67,832	3882	1485	5.72	2.19
	August 29	72,089	4243	1664	5.89	2.31
	August 30	79,789	4679	1721	5.86	2.16
	August 31	88,074	4967	2041	5.64	2.32
	September 1	96,850	5118	2118	5.28	2.19
	September 2	88,455	4770	1975	5.39	2.23
	September 3	99,190	4545	1643	4.58	1.66

^a^*I*: number of impressions.

^b^*C*: clicks.

^c^*V*: page views.

^d^*C/I*: clicks per impression.

^e^*V/I*: page views per impression.

**Table 5:** Results of the ordinary least squares regression of clicks per impression and page views per impression relative to the number of impressions and optimization.

Estimator		Coefficient	SE	*P* value
**Clicks per impression (*C/I*)**				
	Constant	0.0415	0.003	<.001
	Optimization (*O*)	0.0213	0.007	.004
	Impressions (*I*)	−6.977e-07	<0.001	<.001
	Optimization×impressions (O*I)	5.938e-07	<0.001	.003
	*F* statistic (*df*)	85.29 (3,27)	—^a^	<.001
	*R* ^2^	0.905	—	—
	Adjusted *R*^2^	0.894	—	—
**Page views per impression (*V/I*)**				
	Constant	0.0220	0.002	<.001
	Optimization (*O*)	−0.0081	0.005	.10
	Impressions (*I*)	−5.827e-07	<0.001	<.001
	Optimization×impressions (O*I)	<0.001	<0.001	<.001
	*F* statistic (*df*)	25.45 (3,27)	—	<.001
	*R* ^2^	0.739	—	—
	Adjusted *R*^2^	0.710	—	—

^a^Not applicable.

## Discussion

### Principal Findings

In this study, we proposed a framework for a large-scale community-based intervention using social media: SNI. Our framework demonstrated an affordable and effective application of social media in rapidly exposing and engaging large populations to address the disproportionate lack of awareness regarding organ donation among minorities. In a period of 1 month, our SNI was able to engage 1 million individuals, which is a much larger audience compared with traditional community-based interventions focused on health education through more costly and rigid frameworks. These traditional interventions relied heavily on health professional interactions with communities to disseminate generalized information without taking into account specific community information such as demographics, optimally relatable material, or highly shareable content through established social networks. A larger audience in conjunction with tailored content provides an ideal platform to effectively engage a target population while potentiating a shift toward positive attitudes regarding organ donation.

By implicating clicks as a form of positive attitude and engagement with organ donation, we showed that targeting a focused audience with tailored content is key to making an intervention more effective. The higher the number of clicks per impression on certain Web-based materials implied that some content had greater impacts on the target audience in motivating engagement with the material. The most effective content presented to the target audience was automatically learned during the intervention and determined to be an optimization priority. Precisely, 21,000 additional clicks were obtained because of the optimization alone, which shows the efficacy and power of an optimizable data-driven network.

A network-based intervention approach has shown the ability to increase target audience engagement with organ donation compared with traditional community-based approaches. This directly potentiates an increase in the proportion of target audience donors at a particular location. The broader impact of this form of intervention results in network changes that can bolster an established organ donor community with every additional organ donor, leading to a higher clustering coefficient and a decrease in the average path length for organ transplantation within a particular GSN.

### Limitations

The major limitation of our current SNI is its inability to measure the actual donor registrations that were obtained as a direct result of the intervention. Our SNI focused on the efficacy of eliciting a simple behavioral action as a proxy for a shift toward positive attitudes regarding organ donation, namely, a click on the organ donor registration site link.

Another limitation is that the data collected in this study are not recent: the organ donation data from our Twitter sensor were collected from April 2015 to May 2016, and the intervention data from Facebook were collected from August 2016 to September 2016. In the study of organ donation, timely access to longitudinal and high-resolution data on organ donation registrations is a major challenge. Additionally, we only had access to yearly state-level organ registration data obtained from publicly available Donate Life annual reports from 2009 to 2016 [2]. However, we have demonstrated in our results that organ-related tweets are correlated with registration rates at the city level even after controlling for population and additionally validated that the organ-related tweets collected in 2016 increasingly correlate with more recent registration data.

Our results were limited to Hispanics and may not necessarily generalize to other minority populations, such as Asians and American Indians. Future studies will be directed to each specific population with their respective community-driven study designs.

### Conclusions

Organ transplantation remains the only life-saving therapy option for patients with end-stage organ failure. However, the lack of organ donors limits the availability of organs for transplant. Although the numbers of organ donors and transplantations in the United States have doubled over the past 20 years, the demand for organs continues to exceed the supply. In 2016, there were over 30,000 solid organ transplantations; however, more than 120,000 people remain on waiting lists for transplants. Associated health care costs related to the management of end-stage disease and associated disabilities outstrip those of transplantations. Therefore, an increase in awareness is needed particularly among minority populations.

At the center of our intervention is the recognition that sociocultural dynamics greatly affect what people incorporate into their own beliefs. Prior campaigns that successfully addressed minority-related organ donation disparity relied on grassroots initiatives and interventions that addressed social and psychological influences of an inadequate knowledge base, misinformation, and medical distrust [17]. We built upon this community-oriented design by expanding an individual’s social network to incorporate their social media circles. Sociocultural influences and personal experiences have been found to drive engagement with the issue of organ donation during prior grassroots campaigns targeting the African-American minority demographic [17-19]. Taking this into account, we tailored our intervention content to appeal to the target minority population on an intimate level by utilizing personal accounts and relatable statistics while providing the targeted audience with the tools to propagate their newly acquired information within their social context [17].

In this work, we proposed a framework for SNI that is both tailored and large-scale using social media. First, we identified structural disparities in organ transplantation among minority groups using a network-based analysis. Next, we created a digital sensor to monitor population awareness about organ donation using social media and validated the sensor using donation registration data. Afterward, we created an intervention campaign to target a focused audience with educational contents regarding organ donation. Finally, we optimized our SNI to target the contents that were automatically identified as more tailored to our focused audience. Therefore, we proposed a conceptual framework (Figure 1) that puts all these separate pieces together to enable a more systemic approach to effective health literacy interventions.

It is important to note that the network analysis and community detection may be more appropriate for a system-wide evaluation of the UNOS allocation of organs. Instead of measuring individual components of the system such as the proportion of donors at specific locations, network-based analysis can give us systems-level measures such as the average path length. Increasing proportions of donors at individual, possibly disconnected, locations might not necessarily improve the average path length at the system level.

We have shown that social media can be used as a sensor for organ donation awareness. Such a sensor has the potential to monitor organ donation awareness in real time at large-scale. In addition, social media can serve as a platform for delivering large-scale, community-based interventions to raise awareness while improving public attitudes and concern for a public health issue such as organ donation. For future studies, we aim to design a longer SNI capable of capturing changes in organ donation awareness on Twitter because of interventions on Facebook. Likely, these changes will also be associated with organ donation registrations.

## Abbreviations

API: application programming interface
GSN: geographical social network
OPO: organ procurement organization
OLS: ordinary least squares
SNI: social network intervention
UNOS: United Network for Organ Sharing
